# Diagnosis and Treatment of the Several Forms of Rheumatism

**Published:** 1890-03-08

**Authors:** 


### DIAGNOSIS AND TREATMENT OF THE SEVERAL FORMS OF RHEUMATISM.

Dr. Gerhardt, from a comparison of 227 cases of ordinary acute articular rheumatism, 23 of scarlatinal, and 18 of gonorrheal, draws the following conclusions. Acute rheumatism begins in the majority, two-thirds of the cases, in the lower extremity, and in half of these in the ankle or foot.

Antipyrin and salicylic acid were decidedly beneficial in two-thirds of these cases; imperfectly so in one-sixth; and for some reason or other, contra-indicated in another sixth. When, however, one of the drugs seemed undesirable, the other was often given with good results.

Scarlatinal rheumatism showed a preference for the joints of the upper limb, and especially of the hand. These cases were usually of short duration, salicylate of soda acting with promptness.

Gonorrheal rheumatism presents four types:—

1. Synovitis with effusion into both knees, with little or no fever or tendency to implication of the heart, though much to œdema.

2. Obstinate subacute inflammation of several joints, which usually sets in somewhat abruptly with profuse sweats, widespread pains in the limbs, and irregular fever; the fever, however, soon subsides, and some joints may remain unaffected for weeks or months, though it is rare that those of the limbs escape, and when it has lasted for months or years it may induce permanent thickening and stiffness of the joints, with wasting of the muscles.

3. Protracted cases; affecting one joint or but few, though often leading to suppuration.

